# Investigation into the Effects of Backrest Angle and Stick Location on Female Strength

**DOI:** 10.3390/ijerph19010007

**Published:** 2021-12-21

**Authors:** Victor Ei-Wen Lo, Shu-Min Chao

**Affiliations:** Department of Occupational Safety and Health, China Medical University, Taichung City 40604, Taiwan; u104014409@cmu.edu.tw

**Keywords:** aircraft controller, design, maximum strength

## Abstract

Objectives: The purpose of this study was to investigate the effects of backrest angle and hand maneuver direction on maximum hand strength and to recommend a strength value for the hand-controlled stick of an aircraft. Methods: Forty-eight female subjects were recruited to perform simulated forward–backward and adduction–abduction maneuvers using control sticks. Each subject was free from musculoskeletal disorders and pain. The independent variables included four control maneuvers (forward, backward, adduction, abduction), two right-hand control stick locations (central, side), and three backrest angles (90°, 103°, 108°). The dependent variable was maximum hand strength. Results: The maximum strength for forward maneuvers with both central and side sticks was strongest at a 90° backrest angle (*p* < 0.001). The maximum strength for adduction maneuvers with both central and side sticks was also strongest at a 90° backrest angle (*p <* 0.001). On the other hand, the highest strength was observed at a 108° backrest angle when pulling the stick backward (*p <* 0.001). The abduction strength was significantly stronger than the adduction strength with a central stick (*p <* 0.001), but the adduction strength was significantly stronger than the abduction strength with a side stick (*p <* 0.001–*p* = 0.017). The forward and abduction strength were significantly different in different locations (*p <* 0.001). The recommended strength in the Code of Federal Regulations (CFR) by the US FAA is higher than the strength values observed in this study. Conclusions: The backrest angle, directions, and location affected the muscular strength. The recommended values should be reevaluated and adjusted for Taiwanese pilots.

## 1. Introduction

The application of ergonomics in aircraft design is long-standing. Many military aircraft design standards or guidelines are based on the results of ergonomic studies [1,2,3,4,5,6,7]. It may be a potential hazard for the pilots if the force feedback of the controller is too little or too great when they are on duty. Excessive force requirements of flight control is one of the causes of aircraft accidents and errors [8,9]. However, the design guideline for the recommended force only considers male strength and has not been reevaluated or updated in decades. Beringer and colleagues recently published a study report for the Federal Aviation Administration (FAA). Beringer’s studies used 5th percentile female values as the recommended operation strength to guarantee that 95% of the population is able to operate these systems [8,9,10].

Previous studies have shown that there are factors affecting the muscle strength, e.g., ethnic groups, genders, ages, handedness, working postures, and joint angles. As a rule of thumb, female strength is two-thirds the strength of males [11]. In general, the percentage of female to male strength ranges from 45% to 80%, depending on the muscle groups or types of maneuvers [8,9,11,12,13,14,15,16,17,18]. For example, the strengths of females were found to be 53% and 48% of the strength of males for pulling and pushing in sitting positions [14]. A study was conducted by Cheng and Lee (2004) to investigate the effects of pulling types and heights, and their results showed that pulling strengths of females were 59% to 67% of those of males for Taiwanese [13]. 

Furthermore, age also affects strength [19]. A study conducted by Or et al. showed that the pulling strength peaked at the age between 35 and 44 years and declined after 45 years for Chinese people [16]. A similar study was conducted in the U.S., and the results revealed that the pulling strength for males peaked at the age between 35 and 44 years and declined after 45 years for Americans. By comparison, the pulling strength for females reached a maximum at the age of 45–54 years and declined after [18]. As age increases, factors such as the degeneration of the musculoskeletal and motor-neuron systems, decreases in muscle mass, decreases in the elasticity of soft tissue, restrictions in the range of motion of joints, and decreases in daily activities contribute to a decrease in strength [20,21]. A dilemma has existed about the 10% “rules” of differences in the strength between the dominant and non-dominant hands since the 1950s. Recent study results showed that this phenomenon is true for those whose dominant hand is the right hand. However, no such significant difference can be found for left-handed participants [22,23,24]. 

Ethnic group plays an important role in terms of differences in body size, muscle mass, and hand strength [16,25,26,27,28]. For example, the pulling strength of Mexicans is significantly different from that of Americans [29]. Furthermore, compared with the results from international studies, the hand strength of both genders in the manufacturing and healthcare industries in Taiwan was significantly lower (59% to 64%) than that of U.S. and European countries [15,30]. A study conducted by Or et al. showed that the pull strength for American participants was 41.2–91.6% greater than that of Chinese participants [16]. Factors such as genetics, environment, growth background, fitness, and daily exercise habits of military personnel in Taiwan vary from those of other countries, and the design standards/guidelines from other countries may not be applicable to our pilots. Therefore, it is very important to investigate the required force for basic manipulation in order to design suitable controllers for Taiwanese aircraft.

According to previous studies, direction of maneuver is another factor affecting strength [17,31]. Rohmert studied push and pull strength in a standing position and found that the push strength on the right hand was significantly higher than the pull strength [32]. The push and pull strength recommended by the Design Criteria Standard by the Department of Defense in the United States (MIT-STD 1472G, 2012) was calculated in a sitting posture. The recommend pull strength of the right hand is 0.5–6.3 kgw greater than the push strength at different angles of elbow flexion. Right-hand strength in the adduction maneuver is 0.9–3.2 kgw greater than in the abduction maneuver [1]. Laubach studied the strength of pushing forward at three different angles of backrest—103°, 115°, and 155°—and the results revealed that the corresponding strength values were 67.6, 69.6, and 57.1 kgw, respectively. The weakest strength was at a backrest having an angle of 155° [5].

According to previous studies, the backrest angles and directions of maneuvers affect the strength values. The backrest angles used for current trainer and fighter jets are between 90° and 120°. In order to tolerate high G-forces during a dog-fight in a combat situation, the design of the seat backrest angle of 120°, such as that of an F-16, can shorten the vertical distance between the heart and the brain, and thus facilitate the blood supply to the brain. However, this design also restricts the ability of the pilot’s head to rest against the seat when looking straight ahead, and results in a high percentage of neck and muscle pain [33,34]. Therefore, to design the next generation of trainer and combat aircraft for the Taiwanese air force, the establishment of guidelines on the maximum strength value of the hand-controlled stick is essential. The purpose of this study was to investigate the effects of different angles of backrest, locations of the control stick, and maneuver directions on the hand strength and recommend strength value for hand-controlled operations in the cockpit of an aircraft.

## 2. Materials and Methods

This experiment had a within-subject, cross-sectional study design. The three independent factors were force maneuvers (four levels: forward, backward, abduction, adduction), location of control stick for the right hand (two levels: side and center), and backrest angle (three levels: 90°, 103°, 108°). Furthermore, there were only two hand maneuvers for the pilot to control the throttle (forward and backward). In total, there were 26 conditions. With regard to the selection of backrest angle, the backrest angle of 90° is widely used for primary trainers for student aviators. By comparison, the backrest angle of combat jets is between 103° and 120°; for example, the backrest angle is 108° and 120° for the F-35 and F-16, respectively. However, to reduce the neck injury of F-16 pilots, the backrest angle of the new generation of combat jet is less than 109.5°. Therefore, we chose 90° for the trainer jet and 103° or 108° for the next generation combat jet.

### 2.1. Study Subjects

In accordance with the Central Limit Theorem, the sampling data were assumed to follow a normal distribution if the sample size was greater than 30. Therefore, we recruited 48 female subjects. We also performed the Shapiro–Wilk test for normality and failed to reject the null hypothesis (Table 1). Furthermore, according to the requirement to be a qualified pilot proposed by the Air Force Academy, Republic of China (ROCAFA), the height of subjects should be 160–190 cm and their body mass index (BMI) should be 18–28 kg/m^2^. In addition, musculoskeletal disorders or pain reduce strength. The experimenter ensured that each subject was in good health and had experienced no hand injuries, cumulative trauma disorders (CTDs), or musculoskeletal pain within the previous six months.

### 2.2. Instrumentation and Questionnaire

**Figure 1:**
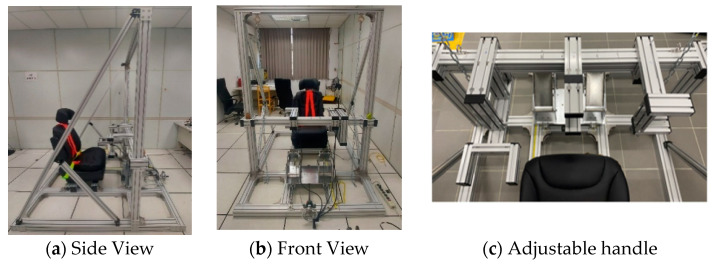
The aluminum fixture simulated the cockpit and stick. (**a**) Side view of the fixture; (**b**) front view of the fixture; (**c**) adjustable handle.

The equipment included an adjustable aluminum fixture with an adjustable power seat and three attached load cells. The dimensions of the aluminum fixture were 2.2 m × 2 m × 2 m (L × W × H) to simulate the aircraft cockpit (Figure 1a,b). The adjustable seat allowed us to change the angle of the backrest and move forward and backward. The adjustable handle (Figure 1c) to simulate the control stick and throttle was attached to a load cell in each direction. This study simulated the T-6B trainer aircraft. The seat of the T-6B trainer aircraft cannot be adjusted. The distance of the rudder paddle is adjustable. The height of the throttle is 57.5 cm from the ground and 36.5 cm from the backrest. The center stick is in the center of the seat. The height of the stick is 73.5 cm from the ground and 54.5 cm from the backrest. The distance between the stick and the throttle is 36 cm. The side stick is 27 cm to the right of the center stick, which is used to simulate the fighting jet. The load cell (LTZ-200KA, Kyowa Electronic Instruments Co., Ltd., Chofu, Japan; LTZ-50KA, Kyowa Electronic Instruments Co., Ltd., Chofu, Japan) was used to measure hand strength. The force data collected by the load cell was transmitted to the computer at a sampling frequency of 100 Hz through a customized system with a multifunctional data acquisition module (USB-6002, National Instruments Co., Austin, TX, USA). 

Demographic variables (e.g., age and sex), anthropometric variables (e.g., height, weight, body mass index, hand width), and lifestyle information (e.g., drinking, smoking, exercise) were collected using a two-page questionnaire.

### 2.3. Experiment Procedures

**Figure 2:**
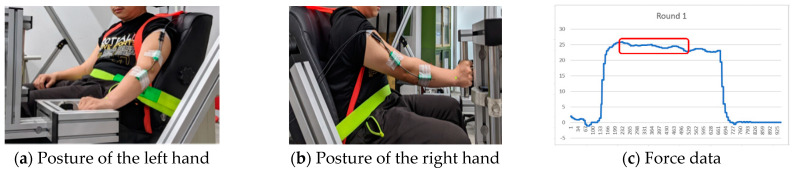
The aluminum fixture simulated the cockpit and stick. (**a**) Posture of the left hand; (**b**) posture of the right hand; (**c**) force data.

The experimenter asked for information on the subjects’ health status when study subjects tried to make an appointment. When the subject arrived, the experimenter checked the biographic information and health status again and then explained the study protocols. Then, the study subject was able to practice for about 5 minutes in order to become familiar with the maneuvers before the formal trials. After the study subjects signed the informed consent form, the experimenter started the trials. The placing of the custom-made horizontal, stainless-steel cylinder on the left-hand side was to simulate the throttle, which only included two maneuvers—push forward and pull backward. The posture is shown in Figure 2a. The setting to simulate the center and side stick control for the right hand is shown in Figure 2b and there were four hand maneuvers—push forward, pull backward, adduction, and abduction.

For each trial, subjects were asked to gradually exert force to their maximum within 1–2 s and maintain that force for 5 s. The mean of the strength data points in the middle of 3 s (Figure 2c) was calculated to represent the maximum strength for each particular trial [35]. Each hand maneuver was measured three times. Then, the mean of the three strength datapoints was calculated to represent the strength for each testing condition. To minimize the effects of fatigue, there were 3 min rest periods between each trial [35]. To ensure data reliability, we calculated the coefficient of variation (CV) based on the data of the three trials. If the value of CV was greater than 10%, the subject was asked to perform the specific hand maneuver again and the experimenter recalculated the CV. Each subject performed right-hand exertions at least 24 times (two locations of control stick × four directions of hand maneuver × three times), and left-hand exertions at least 18 times (two directions of hand force × three times) for each backrest angle. It took 2 h to complete the 42 trails for each backrest angle and there were three backrest angles. In total, study subjects spent at least six hours completing all trials. In order to conveniently set each backrest angle, we treated each backrest angle as a block. Again, to avoid the carry-over effect of muscle fatigue, each subject was limited to 2.5 h of testing per day and had to rest of at least 48 to 72 h between each block. The blocks, and all trials within each block, were randomized and counter-balanced.

### 2.4. Statistical Analysis

The means, standard deviations for continuous variables, and percentages for categorical variables were used to depict the demographic and strength data. The Shapiro–Wilk test was performed to check the normality of the strength data, which was confirmed to be normally distributed. To investigate the effect of the maneuver direction on the hand strength, the paired-t test was performed. Two-way ANOVA was used to compare the effects of backrest angles, stick locations, and maneuvers, and their interactions, on hand strength. The Bonferroni correction was used as a post hoc test. Statistical significance was set at *p* < 0.05. SPSS Chinese version 22.0 (IBM Corporation, Armonk, NY, USA) was used for statistical analyses.

## 3. Results

### 3.1. Demographic Information of Study Subjects

A total of 48 healthy female subjects were recruited in this study. Their demographic and anthropometric information are shown in Table 2. The subjects fulfilled the CAFA requirements (height: 166.3 ± 3.1 cm, weight: 59.0 ± 7.4 kg, BMI: 21.3 ± 2.6 kg/m^2^, age: 23.6 ± 3.2 years). Ninety-four percent of the subjects were right-hand dominant and more than half of the subjects exercised 2–4 times per week (54.2%). Most subjects were students (56.3%), and the remaining subjects worked in manufacturing (12.5%), healthcare (6.2%), service industries (10.4%), or as freelancers (14.6%). All participants were free from any upper-extremity and torso injuries or disorders.

#### 3.1.1. Effect of Backrest Angle on Hand Strength

The effect of backrest angle on hand strength, stick location, and maneuver is depicted in Table 3. Backrest angle had a significant effect on the strength of pulling backward for the left hand, and the strength increased as the backrest angle increased (*p* < 0.001). However, there were no significant differences observed for the push forward strength of the left hand at different backrest angles (*p* = 0.394).

For the right hand, backrest angle had a significant effect on strength for three of the four maneuvers in both stick locations. The only maneuver direction for which the backrest angle did not affect the strength was abduction (*p* = 0.140 for the central stick; *p* = 0.161 for the side stick). For both the side- and center-stick, the strength of pushing forward decreased as the backrest angle increased (all *p* < 0.001). Post hoc testing reveals that the maximum strength for pushing the central and side stick forward was observed at the 90° backrest angle (22.6 ± 4.3 kgw/24.6 ± 5.5 kgw). The maximum strength for adduction in the central- and side-stick positions was also observed at the 90° backrest angle (6.8 ± 1.8 kgw/7.1 ± 1.6 kgw). By comparison, the strength of pulling backward increased as the backrest angle increased (all *p* < 0.001). The maximum strength was observed at 108° of the backrest angle when pulling the stick backward, in both central- and side-sick configurations (29.0 ± 6.6 kgw/28.7 ± 6.2 kgw).

#### 3.1.2. Effect of Maneuvers on Hand Strength

When comparing the strength difference between the different maneuver directions (Table 4), the results revealed that the strength of the pushing and pulling were significantly different (*p* < 0.001) for both hands. The strength of pulling backward (17.3 ± 3.8 kgw) was significantly stronger than pushing forward (15.2 ± 3.4 kgw) with the left hand (*p* < 0.001). For central and side sticks, the strength of pulling backward (26.4 ± 6.8 kgw/26.6±6.4 kgw) was also significantly stronger than pushing forward with the right hand (20.5 ± 4.3 kgw/22.1 ± 5.1 kgw; all *p* < 0.001). For the central stick, the abduction (pulling away from the body) strength (8.4± 1.9 kgw) was significantly stronger than that of adduction (pushing toward the body) (5.7 ± 1.6 kgw; *p* < 0.001). However, the adduction strength (6.4 ± 1.6 kgw) was significantly stronger than the abduction strength (5.6 ± 1.2 kgw; *p* < 0.001) for the side stick.

There were no significant differences in strength between pushing forward and pulling backward for the left hand or for either stick location for the right hand at a backrest angle of 90°. However, the strength of pulling backward was significantly higher than the strength of pushing forward in all trials at backrest angles of 103° and 108°. 

#### 3.1.3. Effect of Stick Locations on Hand Strength

**Figure 3:**
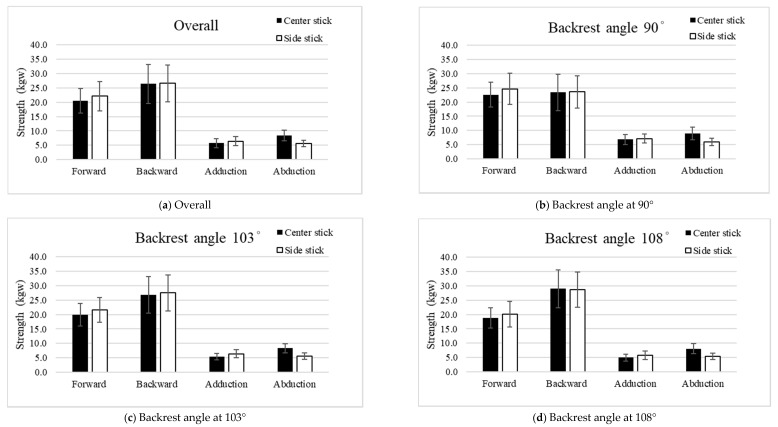
The effects of stick locations on the right-hand strengths at different backrest angles (unit: kgw). (**a**) Overall; (**b**) Backrest angle at 90°; (**c**) Backrest angle at 103°; (**d**) Backrest angle at 108°.

To compare the effects of the stick locations on strength by maneuvers and backrest angle (Figure 3), the results reveal that the strength of the center and side sticks was significantly different in the three maneuver directions (pushing forward, adduction, and abduction). There was no stick location effect on the pulling back strength (26.4 ± 5.8 kgw for the center stick vs. 26.6 ± 5.4 kgw for the side stick; *p* = 0.571).

The strength of pushing forward on the side stick (22.1 ± 5.1 kgw) was significantly stronger than the strength on the center stick (20.5 ± 4.3 kgw; *p* < 0.001). At all three backrest angles, the strength on the side stick was also significantly stronger than the strength on the center stick (*p* < 0.001–*p* = 0.003). The same result was also observed for the adduction maneuver—the strength on the side stick (6.4 ± 1.6 kgw) was significantly stronger than the strength on the center stick (5.7 ± 1.6 kgw; *p* < 0.001). Furthermore, the strength on the side stick was also significantly stronger than the strength on the center stick at the backrest angles of 103° and 108° (all *p* < 0.001). However, the abduction strength on the center stick (8.4 ± 1.9 kgw) was significantly stronger than the strength on the side stick (5.6 ± 1.2 kgw; *p* < 0.001). In addition, the strength on the center stick was also significantly stronger than the strength on the side stick at the three backrest angles (all *p* < 0.001).

#### 3.1.4. Effect of Backrest Angles, Stick Locations and Maneuver Directions on Hand Strength

Table 5 shows the results of the main effects and the interactions between backrest angles (90°, 103°, and 108°), maneuver directions (forward, back, adduction, abduction) and stick locations (central and side) on hand strength. The left-hand strength was significantly affected by the backrest angle (F = 10.792; df = 2; *p* < 0.001) and maneuver direction (F = 24.056; df = 1; *p* < 0.001), and their interaction (F = 3.959; df = 2; *p* = 0.020).

The right-hand strength was significantly affected by the stick location (F = 4.028; df = 1; *p* = 0.045) and maneuver direction (F = 132.948; df = 1; *p* < 0.001), but the backrest angle did not affect the forward–backward strength (F = 0.613; df = 2; *p* = 0.542). In addition, there was an interaction between the effects of backrest angle and direction on the forward–backward strength (F = 39.291; df = 2; *p* < 0.001). The stick location and maneuver direction had a significant main effect on the forward–backward strength (F = 4.028; df = 1; *p* = 0.045 / F = 132.948; df = 1; *p* < 0.001).

Regarding the adduction–abduction strength, there was a significant main effect of backrest angles (F = 23.79; df = 2; *p* < 0.001) and stick location (F = 69.516; df = 1; *p* < 0.001), but the interaction between the backrest angle and the direction did not have a significant effect on the force (F = 39.291; df = 2; *p* < 0.001). In addition, the interaction between the angle of the backrest and the maneuver direction and the interaction between the location and direction also had a significant effect on the force (F = 4.335; df = 2; *p* = 0.014 / F = 191.295; df = 1; *p* < 0.001, respectively).

### 3.2. Recommended Strength and Comparison with the Strength Values Proposed by the FAA

According to previous studies, the strength in both maneuver directions (forward–backward) of the pitch and both maneuver directions (adduction–abduction) of the roll were again averaged to represent the recommended strength value [8,9]. Using the average strength to represent the recommended value may result in a problem whereby the strength is greater than the 5th percentile of female strength in the weaker maneuver direction of the force. In order to avoid overestimating the value, this study used a smaller value for both maneuver directions of the roll and both maneuver directions of the pitch as the recommended value of operation strength (Table 6). 

The upper limit of the control strength was the recommended value based on the strength in the 5th percentile of females. The predicted recommended value of the throttle was 9.1 kgw. The recommended strength values for the center stick in the pitch maneuver and roll maneuver were 12.4 and 3.2 kgw, respectively. The predicted recommended values were 12.8 and 3.5 kgw, respectively, when the control stick was on the side.

## 4. Discussion

Previous studies have shown that backrest angles, maneuver directions, and stick locations affected the hand strength. Laubach investigated the strengths of pushing forward at three different backrest angles (103°, 115°, 155°) and the study results revealed that the strength values were 67.6, 69.6, and 57.1 kgw, respectively, with the pushing forward strength being lowest at a backrest angle of 155°. The pushing forward strength was stronger when the backrest angle was smaller, which is in accordance with this study’s results [5]. When adjusting the backrest angle, subjects also change the angle of the elbow and shoulder in order to grasp the control stick. According to previous studies, the angles of the elbow and shoulder were considered factors that affect the pushing and pulling strength [29,36,37].

Hunsicker (1957) conducted an experiment to study the pushing forward and pulling backward force of aircraft manipulation affected by elbow angles of 60°, 90°, 120°, 150°, and 180°. The strength values of pushing forward, adduction, and abduction maneuvers were highest at an elbow angle of 60° (42.6, 21.8 and 18.6 kgw, respectively) and the strength decreased as the elbow angle increased. By comparison, the degree of elbow flexion had no obvious effect on the pulling backward strength [3]. However, Seo et al. found that the maximum pulling backward force was, on average, 29% greater when the elbow was extended compared to when the elbow was flexed. When the working posture changes, it appears that the extended elbow can reduce the extent of the required joint stabilization, which results in increased maximum pulling backward force [38]. In this study, the extent of the required joint stabilization was reduced as the backrest angle and elbow angle increased. Therefore, the pulling strength increased as the backrest angle (and thus elbow angle) increased, which agrees with the results of Seo et al.’s study.

Previous studies have shown that hand domination affected the strength [22,23,24]. As previously mentioned, the 10% rule can only be applied to those with right-hand domination. Our study results showed that the strength of the pushing maneuver of the left hand was identical for both hands. However, the pulling maneuver of the left hand for right-handed subjects was higher than that for left-handed subjects. By comparison, the strength of right-handed subjects was greater than that of left-handed subjects for all right-hand maneuvers. However, the 95% confidence interval was large due to the small sample size for left-hand dominant subjects (*n*=3), resulting in less-meaningful comparisons. 

Thordsen et al. (1972) conducted a study to simulate aircraft manipulation. The left-hand control stick position was set 33.0 cm in front of the seat reference point and 31.5 cm above the center. Study results revealed that the strength of backward maneuvers was 35.3 kgw, which was significantly greater than the strength of forward maneuvers (31.3 kgw). Furthermore, the strength of adduction was 294.9N (30.1 kgw), which was greater than the strength of abduction, which was 245.6N (25.1 kgw). The result shows that the strength of adduction was greater than the strength of abduction [39]. The study of Cale-Benzoor et al. (2016) found that different muscles were used for pushing (triceps, serratus anterior, anterior deltoid, and scapula extensions) and pulling (triceps long head, biceps, and scapula contractions). The pulling backward force was significantly higher than the pushing forward force when the elbow angle was within 66%–100% of the restricted range of motion [40]. In our study, the elbow position of the right hand forward and backward was greater than 66% of ROM, and the pulling backward force was higher than the pushing forward force. This finding is in accordance with the findings of Cale-Benzoor et al. In the study of La Delfa et al., they showed that the forces were limited by external humeral rotation strength during adduction and abduction maneuvers, and this resulted in the force being smaller in the abduction maneuver [37]. This may explain why the abduction force with the center stick was stronger than that with the side stick in our study.

In Laubach’s study, the position of the center stick was set to 48.0 cm in front of the seat reference point and 38 cm above the seat reference point, while the backrest angle was 103°. The position of the side stick was 13 cm to the right of the center stick. The forward force of the center stick (31.4 kgw) was significantly lower than that of the side stick (35.5 kgw). The result was in accordance with our study results, which show that the pushing forward strength was affected by the location of the control stick [5]. In the Cudlip et al. study, the angles of wrist flexion and extension and an increase in the external rotation of the humerus should result in an increase in the muscular activity in order to achieve the required strength [41]. This was one of the reasons why the strength in the side stick was greater than the strength in the center stick, because the study subjects could maintain the wrist angle in the neutral position when operating the side stick when pushing forward and conducting adduction maneuvers.

Finally, Table 7 shows the comparison between the recommended value from Beringer’s (2019) study, the Code of Federal Regulations (CFR), and the results of this study. The recommended values of the CFR were two to three times greater than the strength values recommended in this study and in Beringer’s studies [8,9]. One of the reasons for this is that the recommended forces in the Code of Federal Regulations (CFR) were based on males’ strength. However, there are more female pilots in the transportation industry and thus it is important to review the recommended values for airplane control. Furthermore, the recommended values in Beringer’s study and the CFR are intended for civil aircraft and may not be valid for military aircraft. Therefore, the testing equipment that simulated aircraft control in those simulations was different from that used in this study. For example, the height of the backrest was lower than the sitting shoulder height and the subjects were not fully supported when pushing forward. This may explain why the recommended values in Beringer’s study were lower than the recommended values in this study.

In comparison to the recommended values for lever operation in US MIL-STD-1472G (2012), the strength value for forward–backward maneuvers was 13.8 kgw, which is higher than the results from our study. In addition, the strength of adduction–abduction maneuvers was 9.2 kgw, which is almost three times higher than our study results [1]. Although gender plays an important role, the different study settings and subjects’ occupations may explain part of the difference. 

This study had three limitations. First, we could not fully simulate the operating conditions of the pilots during flight. For example, the pilots need to operate the aircraft in high G-force conditions, which can restrict the force exertions and result in the pilot being unable to control the aircraft. Furthermore, the pilots also need to exert force using their hands and feet simultaneously in order to control the aircraft. Sharing tasks may also reduce the strength exerted. However, this is the first time that we have tried to understand the strength at different settings in the cockpit in Taiwan. Tests on the effect of dual-control tasks on strength when simulating the operation of an aircraft are ongoing. Second, the loadcell used in this study can only collect the force strength in one direction, and other resultant forces cannot be measured. Finally, the subjects recruited in this study did not have any training or flight control experience. They were not familiar with the maneuvers, which may have resulted in differences in strength between our subjects and professional pilots. Although the recommended strength may be underestimated, it is still valuable because there are increasing numbers of female pilots in the Department of Defense and/or in the aviation industry [8,9,42]. In the future, we will collect the strength data of females with flying experience and increase the number of subjects.

## 5. Conclusions

This was the first study to understand the effects of backrest angle and stick location on flight control by hand in Taiwan. Strength was significantly affected by the backrest angle. In different maneuver directions, the strength of pulling backward was significantly stronger than that of pushing forward. However, the stick location plays a mixed role regarding the strength of adduction–abduction maneuvers. The adduction strength was weaker than the abduction strength with the center stick but was stronger than the abduction strength with the side stick. In the different stick locations, the strength of pushing forward with the side stick was stronger than that with the center stick. No effects of stick location on the pulling backward strength were observed. Finally, the recommended strength in this study was significantly lower than the value recommended by the Code of Federal Regulations (CFR), and this can provide aircraft designers and manufacturers with valuable information that may help to improve aviation safety in the future.

## Figures and Tables

**Table 1:** The results of Shapiro–Wilk test for normality by conditions.

Hand	Stick Location	Maneuver	Backrest Angle (*n* = 48)		
			90°	103°	108°
L	Side	Forward	0.142	0.262	0.358
		Backward	0.237	0.254	0.705
RH	Center	Forward	0.957	0.630	0.381
		Backward	0.422	0.104	0.886
		Adduction	0.168	0.145	0.197
		Abduction	0.118	0.524	0.145
	Side	Forward	0.668	0.275	0.678
		Backward	0.786	0.229	0.433
		Adduction	0.537	0.602	0.125
		Abduction	0.398	0.872	0.474

**Table 2:** Demographic and anthropometric information of the forty-eight female subjects.

Variables		Mean ± SD/*n* (%)
Age (years)		23.4 ± 3.1
Height (cm)		166.3 ± 3.1
Weight (kg)		59.0 ± 7.4
BMI (kg/m^2^)		21.3 ± 2.6
Shoulder–elbow length (cm)		34.2 ± 1.8
Elbow–fingertip length (cm)		43.7 ± 1.7
Forearm circumference (cm)		23.3 ± 1.8
Elbow rest height (cm)		24.1 ± 2.5
Hand length (cm)		17.8 ± 0.8
Hand breadth (cm)		7.7 ± 0.4
Wrist circumference (cm)		14.9 ± 2.0
Dominant (count)	R	45 (93.8%)
	L	3 (6.2%)
Exercise (count)	None	1 (2.1%)
	Sometimes	14 (29.2%)
	1 time per week	4 (8.3%)
	2–4 times per week	26 (54.2%)
	5–7 times per week	3 (6.2%)
Smoking (count)	No	47 (97.9%)
	Quit smoking	1 (2.1%)
	Yes	0 (0.0%)
Drinking (count)	No	36 (75.0%)
	1 time per moth	11 (22.9%)
	1 time per week	1 (2.1%)
	2–3 times per week	0 (0.0%)
	>4 times per week	0 (0.0%)

**Table 3:** The effects of backrest angle on hand strength, stick location, and maneuver (unit: kgw).

Hand	Stick Location	Maneuver	Backrest Angle			*p*-Value ^1^
			90°	103°	108°	
			Mean ± SD	Mean ± SD	Mean ± SD	
LH	Side	Forward	14.7 ± 3.6	15.5 ± 3.3	15.5 ± 3.4	0.394
		Backward	15.2 ± 3.1 ^a^	17.8 ± 3.6 ^b^	18.8 ± 3.7 ^b^	<0.001 **
RH	Center	Forward	22.6 ± 4.3 ^c^	20.0 ± 3.9 ^d^	18.8 ± 3.6 ^d^	<0.001 **
		Backward	23.4 ± 6.4 ^e^	26.8 ± 6.4 ^f^	29.0 ± 6.6 ^f^	<0.001 **
		Adduction	6.8 ± 1.8 ^g^	5.4 ± 1.2 ^h^	5.0 ± 1.2 ^h^	<0.001 **
		Abduction	8.9 ± 2.2	8.3 ± 1.6	8.1 ± 1.8	0.140
	Side	Forward	24.6 ± 5.5 ^i^	21.6 ± 4.3 ^j^	20.1 ± 4.5 ^j^	<0.001 **
		Backward	23.6 ± 5.7 ^k^	27.5 ± 6.2 ^l^	28.7 ± 6.2 ^l^	<0.001 **
		Adduction	7.1 ± 1.6 ^m^	6.4 ± 1.4 ^mn^	5.8 ± 1.5 ^n^	<0.001 **
		Abduction	5.9 ± 1.3	5.6 ± 1.2	5.4 ± 1.1	0.161

^1^ Two-way ANOVA: ** *p <* 0.001. ^a–n^: Same letter denotes that there was no significant difference.

**Table 4:** The effects of maneuvers on the strength by backrest angle and stick location (*n* = 48; unit: kgw).

Hand	Stick Location	Maneuver	Backrest Angle						Total	
			90°		103°		108°			
			Mean ± SD	*p*-Value ^1^	Mean ± SD	*p*-Value ^1^	Mean ± SD	*p*-Value ^1^	Mean ± SD	*p*-Value ^1^
LH	Side	Forward	14.7 ± 3.6	0.236	15.5 ± 3.3	< 0.001 **	15.5 ± 3.4	< 0.001 **	15.2 ± 3.4	< 0.001 **
		Backward	15.2 ± 3.1		17.8 ± 3.6		18.8 ± 3.7		17.3 ± 3.8	
RH	Center	Forward	22.6 ± 4.4	0.332	20.0 ± 3.9	< 0.001 **	18.8 ± 3.6	< 0.001 **	20.5 ± 4.3	< 0.001 **
		Backward	23.4 ± 6.4		26.8 ± 6.4		29.0 ± 6.6		26.4 ± 6.8	
		Adduction	6.8 ± 1.8	< 0.001 **	5.4 ± 1.2	< 0.001 **	5.0 ± 1.2	< 0.001 **	5.7 ± 1.6	< 0.001 **
		Abduction	8.9 ± 2.2		8.3 ± 1.6		8.1 ± 1.8		8.4 ± 1.9	
	Side	Forward	24.6 ± 5.5	0.167	21.6 ± 4.3	< 0.001 **	20.1 ± 4.5	< 0.001 **	22.1 ± 5.1	< 0.001 **
		Backward	23.6 ± 5.7		27.5 ± 6.2		28.7 ± 6.2		26.6 ± 6.4	
		Adduction	7.1 ± 1.6	< 0.001 **	6.4 ± 1.4	< 0.001 **	5.8 ± 1.5	0.017 *	6.4 ± 1.6	< 0.001 **
		Abduction	5.9 ± 1.3		5.6 ± 1.2		5.4 ± 1.1		5.6 ± 1.2	

^1^: Paired Sample *t* test: * *p <* 0.05; ** *p <* 0.001.

**Table 5:** The strength of the effects of the different of backrest angles, stick locations, and maneuver directions.

Hand	Maneuver Direction	Source	Sum of Squares	Degree of Freedom	Mean Square	F	*p*-Value
Left	Forward-backward	Backrest angle	259.121	2	129.56	10.792	<0.001 **
		Maneuver direction	288.801	1	288.801	24.056	<0.001 **
		Backrest angle × Maneuver direction	95.051	2	47.525	3.959	0.02
		Error	3385.57	282	12.006		
Right	Forward-backward	Backrest angle	35.883	2	17.941	0.613	0.542
		Stick location	117.903	1	117.903	4.028	0.045 *
		Maneuver direction	3891.68	1	3891.68	132.948	<0.001 **
		Backrest angle × Stick location	14.406	2	7.203	0.246	0.782
		Backrest angle × Maneuver direction	2300.26	2	1150.13	39.291	<0.001 **
		Stick location × Maneuver direction	77.587	1	77.587	2.651	0.104
		Backrest angle × Stick location × Maneuver direction	4.951	2	2.476	0.085	0.919
		Error	16509.5	564	29.272		
	Adduction-abduction	Backrest angle	112.174	2	56.087	23.797	<0.001 **
		Stick location	163.84	1	163.84	69.516	<0.001 **
		Maneuver direction	124.322	1	124.322	52.749	<0.001 **
		Backrest angle × Stick location	5.439	2	2.719	1.154	0.316
		Backrest angle × Maneuver direction	20.433	2	10.217	4.335	0.014 *
		Stick location × Maneuver direction	450.854	1	450.854	191.295	<0.001 **
		Backrest angle × Stick location × Maneuver direction	0.97	2	0.485	0.206	0.814
		Error	1329.27	564	2.357		

* *p* < 0.05; ** *p* < 0.001.

**Table 6:** Comparison between the FAA recommended one-hand control force with the female strength in this study (unit: kgw).

Flight Control		Backrest Angle									Overall			FAA 14 CFR 23.146 (c)
		90°			103°			108°						
		5th	25th	50th	5th	25th	50th	5th	25th	50th	5th	25th	50th	Recommended
Throttle	LH	9.1	12.1	14.6	10.5	13.1	15.2	10.0	13.3	15.0	9.1	12.1	14.6	-
Center stick	Pitch	12.4	18.8	22.8	14.1	17.5	19.5	13.4	16.0	17.9	12.4	16.0	17.9	22.7
	Roll	4.3	5.2	6.8	3.5	4.4	5.4	3.2	4.2	4.9	3.2	4.2	4.9	11.4
Right stick	Pitch	14.7	20.2	23.2	14.7	18.2	22.4	12.8	17.3	20.2	12.8	17.3	20.2	-
	Roll	3.8	5.0	5.6	3.5	4.7	5.6	3.6	4.6	5.3	3.5	4.6	5.3	-

(c) is the FAA guide line.

**Table 7:** The recommended stick strength values (unit: kgw).

Studies	Gender	Center stick		Right stick	
		Pitch	Roll	Pitch	Roll
FAA 14CFR	Male	22.7	11.4	N/A	N/A
Beringer (2019)	Female	5.9	3.2	6.4	2.7
This Study	Female	12.4	3.2	12.8	3.5

## Data Availability

The data presented in this study are available on request from the corresponding author. The data are not publicly available due to privacy or ethical concerns.

## Abbreviations

CFR	Code of Federal Regulations
FAA	Federal Aviation Administration
BMI	Body mass index
CTDs	Cumulative trauma disorders
CV	Coefficients of variation
ANOVA	Analysis of variance
